# Integrating serum pharmacochemistry and network pharmacology to explore potential compounds and mechanisms of Alpiniae oxyphyllae fructus in the treatment of cellular senescence in diabetic kidney disease

**DOI:** 10.3389/fmed.2024.1424644

**Published:** 2024-07-03

**Authors:** Zijie Yan, Lin Zhang, Yu Kang, Shuman Liu, Xiaoyan Li, Lidan Li, Kai Rui, Man Xiao, Yiqiang Xie

**Affiliations:** ^1^College of Traditional Chinese Medicine, Hainan Medical University, Haikou, China; ^2^Heilongjiang Academy of Traditional Chinese Medicine, Harbin, China; ^3^First Clinical College of Medicine of Guizhou University of Traditional Chinese Medicine, Guiyang, China; ^4^Key Laboratory of Biochemistry and Molecular Biology, Hainan Medical University, Haikou, China

**Keywords:** diabetic kidney disease, traditional Chinese medicine, cellular senescence, Alpiniae oxyphyllae fructus, UHPLC-QTOF-MS, network pharmacology, molecular docking

## Abstract

**Background:**

Diabetic kidney disease (DKD), one of the microvascular complications in patients with diabetes mellitus, is a common cause of end-stage renal disease. Cellular senescence is believed to be an essential participant in the pathogenesis of DKD. Although there is evidence that Alpiniae oxyphyllae fructus (AOF) can ameliorate DKD progression and organismal senescence, its ability to ameliorate renal cellular senescence in DKD as well as active components and molecular mechanisms remain to be explored.

**Purpose:**

This study aimed to investigate the role of AOF in the treatment of cellular senescence in DKD and to explore its active components and potential molecular mechanisms.

**Methods:**

The pharmacological efficacy of AOF in ameliorating cellular senescence in DKD was assessed by establishing DKD mouse models and HK-2 cells under high glucose stress. UHPLC-QTOF-MS was used to screen the active compounds in AOF, which were used in conjunction with network pharmacology to predict the molecular mechanism of AOF in the treatment of cellular senescence in DKD.

**Results:**

*In vivo* experiments showed that AOF reduced GLU, mAlb, Scr, BUN, MDA, SOD levels, and ameliorated renal pathological damage and renal cell senescence in DKD mice. *In vitro* experiments showed that AOF-containing serum improved the decline in HK-2 cell viability and alleviated cellular senescence under high glucose intervention. The results of the UHPLC-QTOF-MS screened 26 active compounds of AOF. The network pharmacological analyses revealed that Cubebin, 2′,6′-dihydroxy-4′-methoxydihydrochalcone, Chalcone base + 3O,1Prenyl, Batatasin IV, and Lucidenolactone were the five core compounds and TP53, SRC, STAT3, PIK3CA, and AKT1 are the five core targets of AOF in the treatment of DKD. Molecular docking simulation results showed that the five core compounds had good binding ability to the five core targets. Western blot validated the network pharmacological prediction results and showed that AOF and AOF-containing serum down-regulate the expression of TP53, and phosphorylation of SRC, STAT3, PIK3CA, and AKT.

**Conclusion:**

Our study shows that AOF may delay the development of cellular senescence in DKD by down-regulating the levels of TP53, and phosphorylation of SRC, STAT3, PIK3CA, and AKT.

## 1 Introduction

Diabetes mellitus (DM) is one of the fastest-growing global health emergencies of the 21^st^ century. The latest data from the 10th edition of the International Diabetes Federation (IDF) Diabetes Atlas shows that the number of people with DM worldwide is expected to increase to 783 million in 2045 (1). Diabetic kidney disease (DKD), one of the microvascular complications in patients with DM, is a common cause of end-stage renal disease. These patients have the highest morbidity and mortality compared to patients with other diabetic complications or chronic kidney disease (2).

Cellular senescence, defined as the entry of cells into a state of permanent cell cycle arrest, plays a vital role in the progression of DKD (3). The findings showed that a large number of senescent cells are observed in renal tissues from DKD patients (4) and that senescence and damage occur in renal proximal tubular epithelial cells (5), glomerular thylakoid cells (6), podocytes (7), and endothelial cells (8) that are exposed to the diabetic environment. Additionally, the gene targeting or administration of Senolytics drugs resulted in improved glucose tolerance, increased insulin sensitivity, and reduced circulating inflammatory mediators in diet-induced and genetically induced obese mice. The anti-aging treatment also improved microalbuminuria, renal podocyte function, and cardiac diastolic function (9). Therefore, therapies targeting renal cellular senescence may become significant in slowing the progression of DKD.

Current clinical therapies for DKD recommend medications that control hypertension and hyperglycemia, such as the renin-angiotensin-aldosterone system inhibitors, sodium-dependent glucose transporter 2 (SGLT-2) inhibitors, or glucagon-like peptide 1 receptor agonists (10). However, drugs such as SGLT2 inhibitors may have side effects such as genitourinary tract infections, lower limb amputations, diabetic ketoacidosis, and hypoglycemia, limiting their clinical application (11), which has led to the exploration of alternative therapies, such as the use of natural botanicals.

According to the Chinese pharmacopeia and flora of China, Alpiniae oxyphyllae fructus (AOF, Yizhiren in Chinese), the dried and ripe fruits of *Alpinia oxyphylla* Miq., is a herbal medicine with a homology of medicine and food. It is mainly distributed in Guangdong, Hainan, and Guangxi in China and has the effect of warming the kidney, consolidating the essence, and reducing urine. Modern pharmacological studies have shown that AOF has various pharmacological activities, such as anti-diuretic, anti-inflammatory, antioxidant, and anti-diabetic activities (12). Previous clinical studies conducted by our group have found that Chinese medicine compounds consisting of AOF as the monarch drug have good clinical efficacy including slowing down renal injury and controlling urinary protein in DKD patients (13–15). It has also been found that AOF extract could reduce blood glucose and protect against renal impairment in DKD model mice (16, 17). Furthermore, we found that AOF could mitigate age-related phenotypes, improve resistance to thermal and oxidative stress, and prolong the lifespan in Caenorhabditis elegans (18). Therefore, we speculate that AOF can be used for the treatment of cellular senescence DKD, but its active compounds and mechanisms remain to be explored further.

The composition of traditional Chinese herbal medicine is complex and those components of drugs that are absorbed into the blood are considered active compounds that exert their medicinal effects (19). Ultra-high performance liquid chromatography-quadrupole time-of-flight mass spectrometry (UHPLC-QTOF-MS) with high resolution, high sensitivity, and high accuracy can provide accurate relative molecular mass and molecular structure, and is widely used for the screening and identification of chemical components in traditional Chinese herbal medicine (20). Network pharmacology is a comprehensive discipline that combines systems biology, information network science, and pharmacology (21). By constructing a drug-target-disease network, it is possible to discover the biological functions of the nodes throughout the network and their interactions with disease. The screening of the incoming serum components of drugs by UHPLC-QTOF-MS and combining it with network analysis can provide new insights into the screening and mechanistic exploration of the active compounds of drugs, which largely accelerates the modernization and internationalization process of traditional Chinese medicine (TCM).

**Figure 1:**
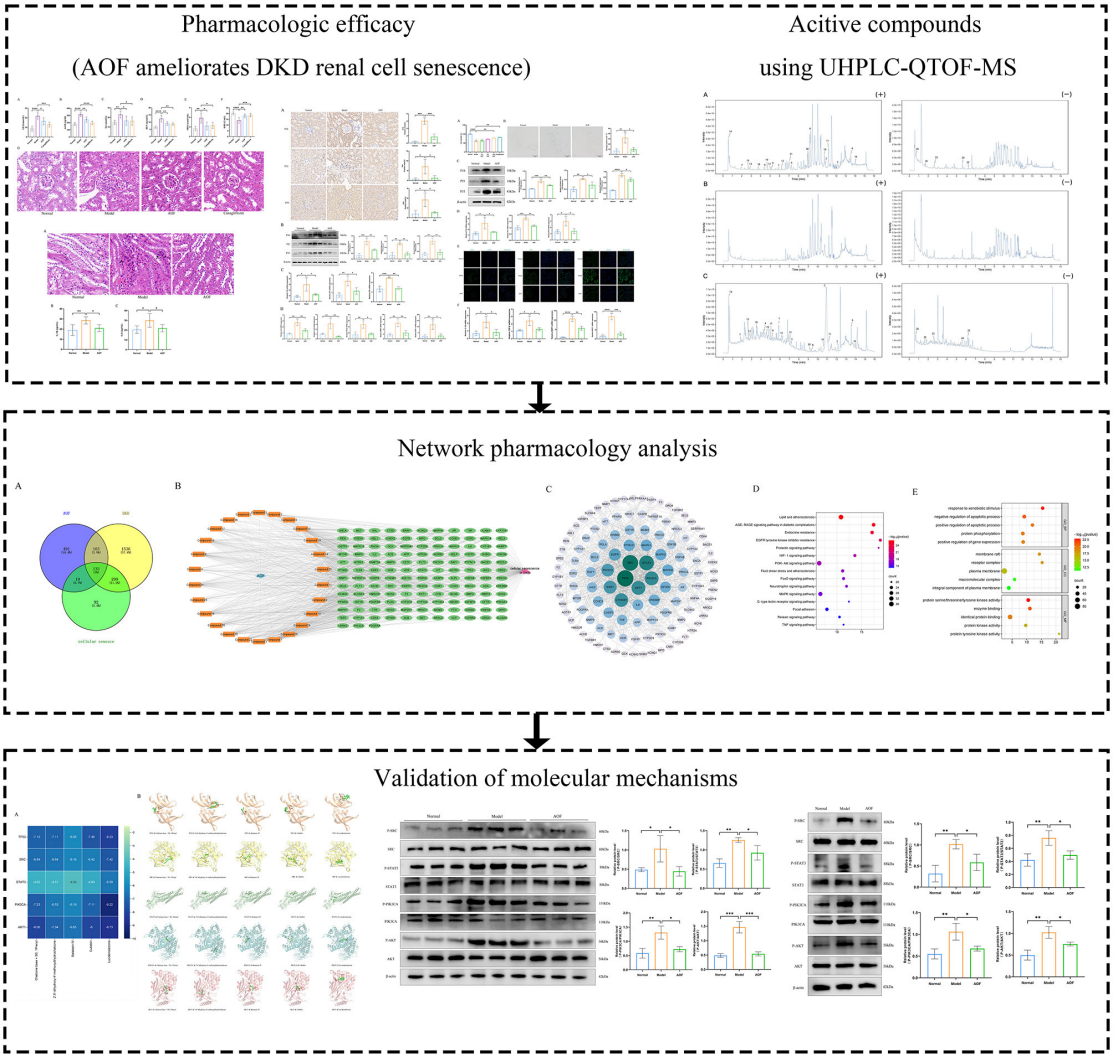
The brief flow chart of this study. In this paper, the efficacy of AOF in ameliorating renal cellular senescence in DKD was firstly confirmed by *in vitro* and *in vivo* experiments, then the active compounds of AOF and their potential molecular mechanisms were identified by UHPLC-QTOF-MS analysis and network pharmacology analysis, and finally the predicted results of network pharmacology were verified by molecular docking and western blot.

In this study, we evaluated the therapeutic effect of AOF on cellular senescence in DKD by establishing DKD renal proximal tubular epithelial cells and mice models. Then, we screened the serum entry components of AOF using UHPLC-QTOF-MS and explored the potential mechanism of AOF in the treatment of DKD cellular senescence in combination with network pharmacological analysis. Finally, the predicted results of network pharmacology were validated by molecular docking simulation and *in vivo* and *in vitro* experiments. The study flow chart is shown in Figure 1.

## 2 Material and methods

### 2.1 Reagents and materials

Alpiniae oxyphyllae fructus (Tongrentang Long Kunnan store, Hainan, China) was identified by Professor Yiqiang Xie, College of Traditional Chinese Medicine, Hainan Medical University; LC-MS grade Methano, Acetonitrile, Formic acid (Thermo scientific, USA); 2-Chloro-L-phenylalanine (purity ≥ 98%) (Hengbai Biotechnology Ltd., Shanghai, China). Canagliflozin (the First Affiliated Hospital of Hainan Medical University, Hainan, China); Creatinine (Cr) Assay kit, blood urea nitrogen (BUN) assay kit, Malondialdehyde (MDA) assay kit, Superoxide Dismutase (SOD) assay kit (Nanjing Jiancheng. Nanjing, China); Eastep^®^ Super Total RNA Extraction Kit (Promega, Shanghai, China); Hifair^®^II1st Strand cDNA Synthesis Kit, Hifair^®^ II 1st Strand cDNA Synthesis SuperMix, Alexa Fluor 488 AffiniPure Rabbit Anti-Goat IgG(H+L) (Yeasen, Shanghai, China); BCA Protein Quantification Kit, RIPA Lysis Buffer, Phenylmethanesulfonyl fluoride (PMSF), HRP-labeled Goat Anti-Rabbit IgG, Senescence β-Galactosidase Staining Kit (Beyotime. Shanghai, China); Mouse microalbuminuria (mAlb) ELISA Kit, Mouse IL-1β ELISA Research Kit, Mouse IL-1β ELISA Research Kit (FANKEW. Shanghai, China); SDS-PAGE Preparation kit (Sangon, Shanghai, China); P16, P21, P53, P-STAT3, P-PIK3CA antibody (Affinity, Jiangsu, China); PIK3CA, STAT3 antibody (Huabio, Zhejiang, China); AKT antibody, P-AKT antibody (MCE, Shanghai, China); P-SRC, SRC antibody (Cell Signaling Technology, Shanghai, China); Anti-fluorescence quenching encapsulant (containing DAPI), β-actin antibody (Servicebio, Wuhan, China).

Six-week-old Sprague Dawley (SD) rats, 6-week-old congenital gene-deficient db/db mice (C57BLKS/J), and 6-week-old wild-type db/m littermates were purchased from GemPharmatech Co., Ltd. (Nanjing, China). All mice were acclimatized to the laboratory for 2 weeks before carrying out the experiment. The laboratory is a specific pathogen-free barrier facility with constant humidity (50% ± 5%) and temperature (22°C ± 2°C) and a 12 h light/dark cycle. All the mice were fed a standard diet and provided with water *ad libitum*. All animal studies were conducted by the Guide for the Care and Use of Laboratory Animals and approved by the Animal Care and Ethics Committee of Hainan Medical University (approval ID: HYLL-2021-389).

### 2.2 Preparation of extracts and drug-containing serum of AOF

The decoction of AOF is prepared by mixing AOF and sterile water. First, the tablets of AOF were placed in a container, mixed with sterile water (eight times the weight of AOF by the weight of water), and steeped for 0.5 h. The mixture is heated for 1 h and filtered to collect the filtrate. The process is repeated twice to obtain a consolidated decoction. The final decoction was concentrated and stored at −20 °C. The final concentration was 1 g/ml. All SD rats received drug treatment after 1 week of acclimatization feeding. The standard dose of AOF extract was 9 g/kg/day per rat based on body surface area (10 times the converted dose based on the body surface area of adults and rats), and the normal group received an equivalent amount of saline. Gavage was administered once a day for 7 days. Blood was taken from the abdominal aorta under anesthesia 2 h after the last gavage. The blood was allowed to stand for 2 h and then centrifuged at 3,000 rpm for 10 min to extract the upper layer of serum, which was dispensed into 1.5 mL sterile EP tubes, inactivated in a water bath at 56°C for 30 min, and stored at −80°C for subsequent experiments.

### 2.3 UHPLC-QTOF-MS analysis

#### 2.3.1 Metabolites extraction of TCM sample

The AOF sample was added to the extract containing the internal standard and homogenized and sonicated in an ice water bath. After 1 h at −20°C, the sample was centrifuged at 4°C/12,000 rpm for 15 min. Finally, the supernatant was removed and placed in a new EP tube for subsequent LC-MS/MS analysis.

#### 2.3.2 Metabolites extraction of serum sample

Serum samples are added to hydrochloric acid and the mixture is vortexed for 1 min and then incubated at 4°C for 15 min. Repeat 4 times. Acetonitrile was then added and the mixture was vortexed for 5 min and centrifuged at 12,000 rpm for 5 min at 4°C. The supernatant was transferred to a new tube. The supernatant was transferred to a new tube and dried. The dried sample is reconstituted by vortexing in methanol containing the internal standard for 5 min. The sample is then centrifuged at 4°C/12,000 rpm for 5 min and transferred to a new container for subsequent LC-MS/MS analysis.

#### 2.3.3 Data processing

Using XCMS software to import mass spectra for analysis. Perform retention time correction, peak identification, peak extraction, peak integration, and peak alignment. Using the secondary mass spectrometry database and the corresponding cleavage pattern matching method, the peaks containing MS data were identified.

### 2.4 Network pharmacology analysis

In this study, we imported compounds obtained from mass spectrometry analysis into Pubchem (https://pubchem.ncbi.nlm.nih.gov/) and structures obtained from Pubchem into the Swisstarget Prediction database (http://www.swisstargetprediction.ch/) as well as the phamMapper database (http://www.lilab-ecust.cn/pharmmapper/) for drug-related target prediction. DKD-related target genes were obtained from the Online Mendelian Inheritance in Humans (OMIM) (http://www.omim.org) and genecards (https://www.genecards.org/) databases. Drug-disease cross-targets were obtained through Venny 2.1.0 (https://bioinfogp.cnb.csic.es/tools/venny/index.html) and cross-targets were imported into the STRING database (https://string-db.org/) to obtain protein-protein interaction (PPI) profiles, then “active compounds-therapeutic targets” network was visualized and mapped using Cytoscape 3.9.1 software. Cross-targets were imported into the DAVID database (https://david.ncifcrf.gov/) for gene ontology (GO) enrichment analysis and Kyoto Encyclopedia of Genomes (KEGG) pathway analysis. The enrichment results were output as bubble plots using the online web bioinformatics (https://www.bioinformatics.com.cn/).

### 2.5 Molecular docking

The crystal structures of proteins were retrieved from the Protein Data Bank (PDB) (PDB; http://www.rcsb.org/pdb/). The water molecules and small molecule ligands of the protein were removed by PyMOL software, and the protein was subjected to hydrogenation operations by AutoDock Vina 1.1.2. Structure files of small molecule compounds with sdf format were obtained from the Pubchem database and converted to mol2 format using Open Bable GUL. Components and targets were converted to PDBQT format files by AutoDock Vina 1.1.2 and molecular docking was performed to measure the interactions between small molecule compounds and proteins. After molecular docking, the conformation with the lowest binding energy was selected as the binding conformation between the ligand and the target protein. Finally, the ligand-protein complexes were analyzed and visualized using PyMOL software.

### 2.6 Animal experiments

#### 2.6.1 Experimental protocol

The mice were randomly divided into four groups and treated as follows: (1) Normal group: db/m mice, received only the same dose of sterilized water as the drug once a day for 8 weeks; (2) Model group: db/db mice, received only the same dose of sterilized water as the drug once a day for 8 weeks; (3) AOF group: db/db mice, received 1.25 mg/g/day AOF extract once a day for 8 weeks. The equivalent dosage of AOF was determined based on the clinical daily dose (10 g AOF/70 kg person) according to the dose normalization by body surface area; (4) Canagliflozin group: db/db mice, received 12.5 mg/kg/day Canagliflozin, once a day for 8 weeks. The equivalent dosage of Canagliflozin was calculated based on the clinical daily dose (0.1 g Canagliflozin/70 kg person) according to the dose normalization by body surface area.

#### 2.6.2 Biochemical index analysis

Before the last day of administration, mice were tested for random blood glucose (GLU) and 24-h urine was collected for urinary microalbumin (mAlb). All mice were anesthetized by inhalation after fasting overnight and then blood samples were collected. To obtain serum samples, blood samples were centrifuged at 3,000 rpm for 15 min and then stored at −80°C until use. Serum creatinine (Scr) and blood urea nitrogen (BUN) were measured using the corresponding kits.

#### 2.6.3 Histopathological examination

After the execution of the mice, both kidneys were removed. Half of the kidneys were immediately frozen in liquid nitrogen for subsequent extraction of RNA and protein, and the remaining kidneys were cut into transverse slices and fixed in 4% paraformaldehyde. After fixation with paraffin wax, hematoxylin and eosin (HE) staining was performed, followed by observation and photography using light microscopy.

#### 2.6.4 Immunohistochemical staining

Kidney tissue samples were deparaffinised to inactivate endogenous peroxidase and subjected to antigen repair, containment, primary and secondary antibody incubation, chromatography, re-staining, and images were obtained using microscopy. Finally, positive areas were calculated using ImageJ software.

#### 2.6.5 IL-1β and IL-6 contents assay

An equal amount of kidney tissue samples were taken and added to PBS and ground in a cryomill at −40°C. Then the supernatant was extracted after centrifugation at 3,000 rmp for 10 min, and the expression levels of IL-6 and IL-1β were detected by using the instructions of the corresponding ELISA kits.

### 2.7 Cell experiment

#### 2.7.1 Cell culture and grouping

HK-2 cells were maintained in an incubator with MEM medium containing 10% FBS or rat serum at 5% CO_2_ and 37°C. Cells were divided into five groups: (1) Normal group; (2) Model group (60 mmol/L glucose); (3) AOF low dose (LAOF) group (60 mmol/L glucose, 2.5% AOF); (4) AOF medium dose (MAOF) group (60 mmol/L glucose, 5% AOF); and (5) AOF high dose (HAOF) group (60 mmol/L glucose, 10% AOF).

#### 2.7.2 CCK8 assay

The 96-well plates were inoculated with suspension of HK-2 cells (100 ul/well). After the molding and drug treatment, the waste solution was aspirated, 10 ul CCK8 solution and 100 ul medium were added to each well, incubation was continued in a cell incubator at 37°C, and the absorbance value at 450 nm was measured by an enzyme marker.

#### 2.7.3 SA-β-gal staining

The staining procedure was carried out according to the instructions of the Senescence β-Galactosidase Staining Kit. Then it was observed and imaged under a light microscope. Blue staining is believed to be an area of accumulation in senescent cells.

#### 2.7.4 Immunofluorescence staining

HK-2 cells inoculated in 24-well plates were subjected to modeling and drug intervention. They were washed three times with PBS before staining, then fixed with 4% paraformaldehyde and permeabilized with 0.2% Triton-100. Next, after blocking with 10% goat serum, the cells were incubated with primary antibody at 4°C overnight. The next day, the sections were incubated with secondary antibody for 1 h at room temperature. Finally, an anti-fluorescence quenching blocking solution (containing DAPI) was added and photographed under a fluorescence microscope.

### 2.8 Reverse transcription-real-time quantitative PCR

Total RNA was isolated from kidney tissues of mice or cell samples, tested for purity and concentration using a Nanodrop 2000 spectrophotometer, and reverse-transcribed into cDNA. A PCR system was prepared for quantitative PCR amplification. Relative mRNA expression levels were normalized to β-actin expression levels. The RT–qPCR results were calculated using the 2^−Δ*ΔCt*^ method. The Primer pairs used in this study were synthesized by Tsingke Biotechnology Co., Ltd. (Beijing, China). The sequences of primers are shown in Supplementary Table 1.

### 2.9 Western blot

First, the kidney tissues of mice or cells were lysed using RIPA and PMSF to obtain total protein samples. The total protein concentration was determined using the BCA protein quantification kit to obtain consistent protein concentrations. Protein samples were separated on SDS-PAGE gels and transferred to polyvinylidene difluoride (PVDF) membranes. PVDF membranes were then sealed with 5% skim milk for 2 h at room temperature and left overnight at 4°C with P16, P21, P53 primary antibody solutions. The PVDF membranes were incubated for 1 h at room temperature using secondary antibodies. Finally, the grayscale values of each imaged protein band were analyzed using a gel imaging system. The protein expression levels were analyzed using β-actin as an internal reference.

### 2.10 Statistical analysis

Experimental data are expressed as mean ± standard deviation. Statistical analyses of multiple groups of samples were performed using a one-way analysis of variance (ANOVA). The Tukey method was used to compare any two groups of data. All statistical data were analyzed using GraphPad Prism 9.4.1. *P* < 0.05 was considered statistically significant.

## 3 Results

### 3.1 AOF improves renal function and pathological tissue damage in DKD mice

**Figure 2:**
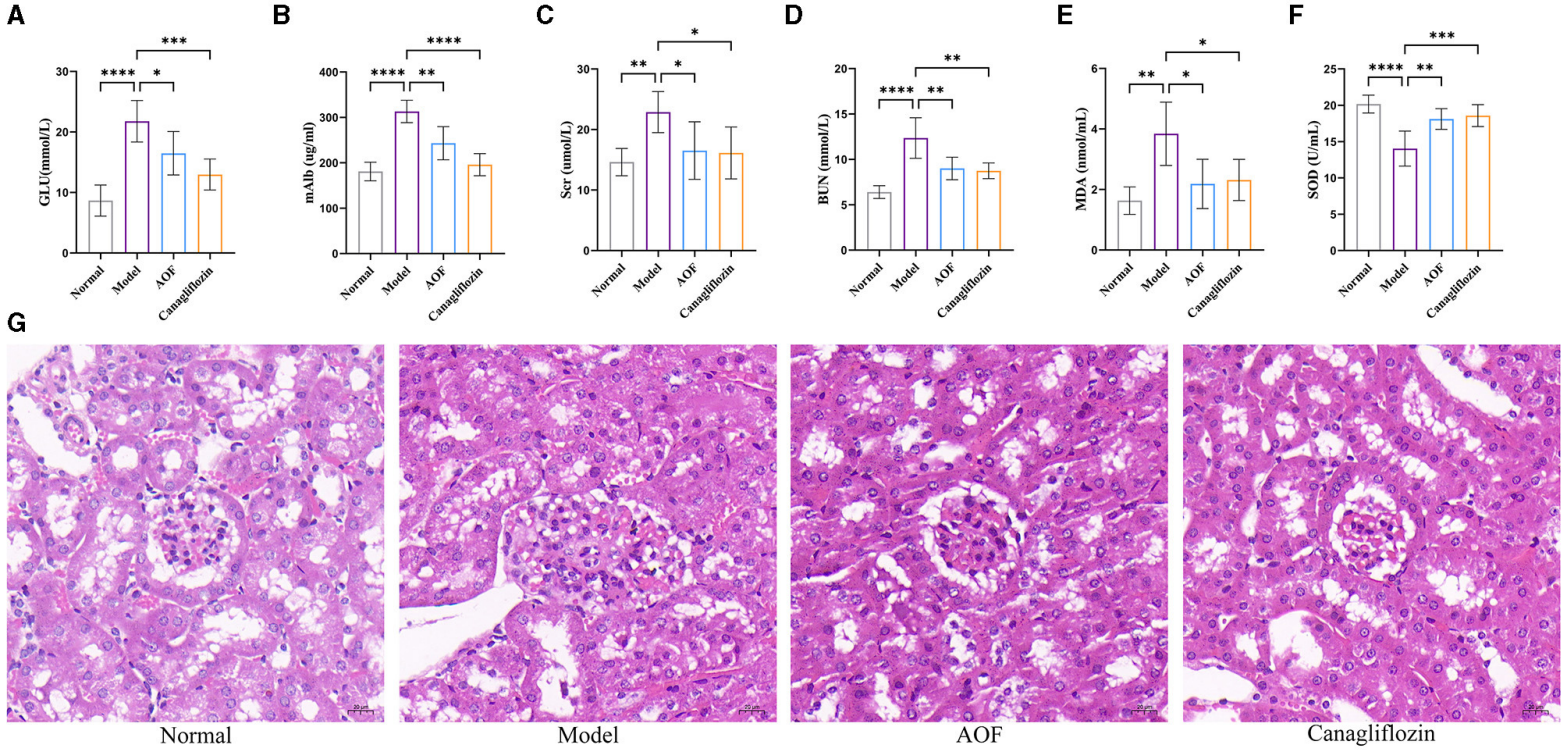
AOF ameliorated biochemical indices and kidney histopathology in DKD mice. Effect of AOF on the GLU **(A)**, mAlb **(B)**, Scr **(C)**, BUN **(D)**, MDA **(E)**, SOD **(F)** after treatment. Histopathological changes in kidney tissues evaluated by HE staining (original magnification × 40.0) **(G)**. The scale bar represents 20 μm. *P < 0.05, **P < 0.01, ***P < 0.001 and ****P < 0.0001.

The levels of GLU, mAlb, Scr, BUN, MDA, and SOD were significantly higher in the model group than in the normal group. After the therapeutic intervention with AOF and canagliflozin, the biochemical indices of the DKD mice showed different degrees of reduction, as shown in Figures 2A–F. HE staining showed that the glomeruli and tubules in the normal group were structurally intact and morphologically clear, specifically the glomerular capsule cavity was clearly visible, the basement membrane and stroma were not significantly hyperplastic, and the tubules were regularly arranged. Compared with the normal group, the glomeruli in the model group had an increased swollen state, thickened basement membrane, swollen tubular epithelial cells and narrowed tubular lumen. After drug intervention, the renal pathological changes in each administration group were reduced to different degrees in both the AOF and Canagliflozin groups, as shown Figure 2G.

### 3.2 AOF ameliorates cellular senescence in the kidney of DKD mice

**Figure 3:**
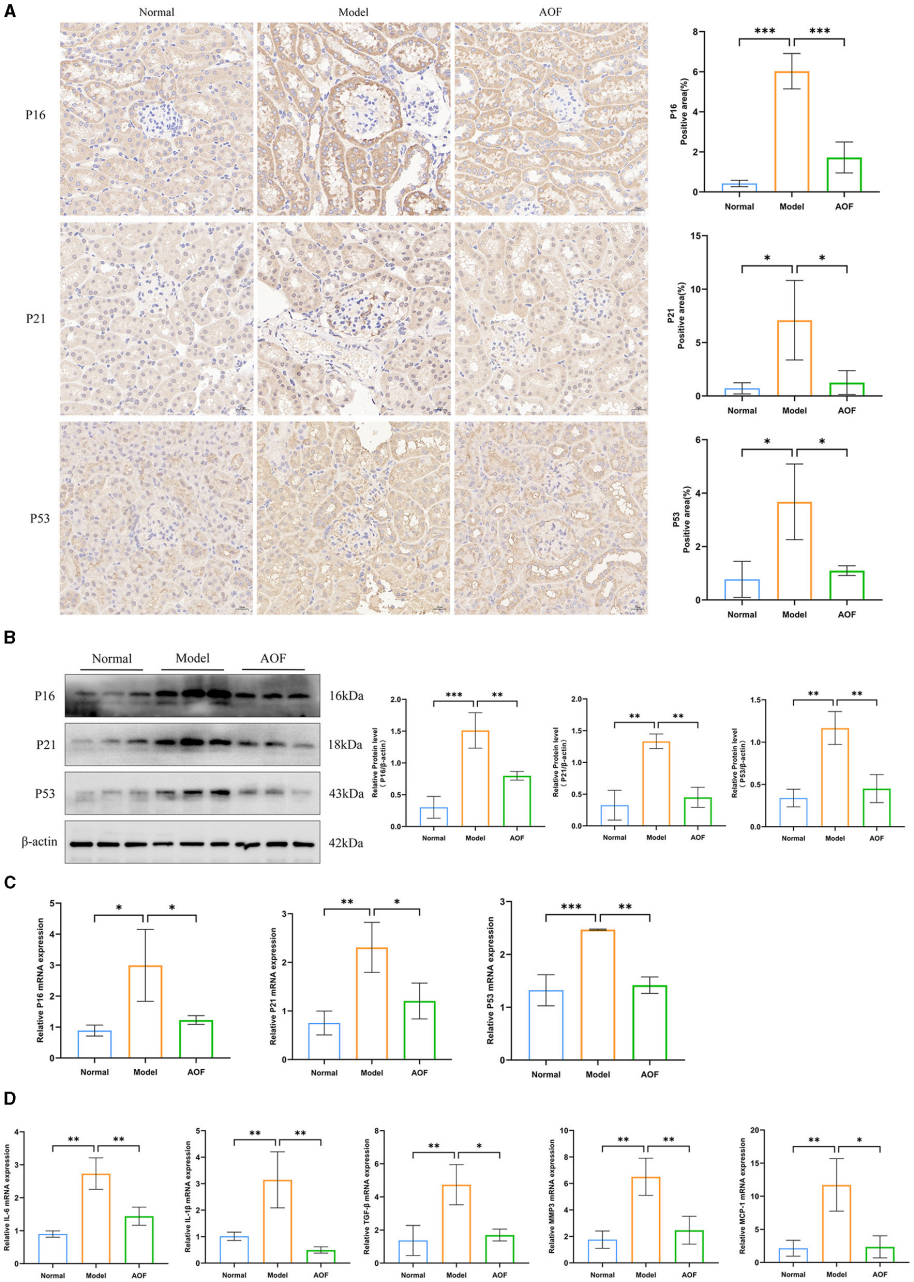
AOF ameliorates cellular senescence in the kidney of DKD mice. Immunohistochemical staining images of P16, P21, P53 **(A)**; P16, P21, P53 protein expression levels **(B)**. P16, P21, P53 mRNA expression levels **(C)**; IL-6, IL-1β, TGF-β, MMP3, MCP1 mRNA expression levels **(D)**. *P < 0.05, **P < 0.01, ***P < 0.001.

Immunohistochemistry showed that the positive expression levels of P16, P21, and P53 in the kidneys of the normal group were lower than those in the model group, and the positive expression levels of P16, P21, and P53 in the kidneys of the model group were higher than those in the AOF group, as shown in Figure 3A. RT-qPCR and western blot results showed that the expression levels of P16, P21, P53 mRNA, and protein were significantly higher in kidneys of the model group compared with those of the normal group, while the expression levels of P16, P21, P53 mRNA, and protein were lower after AOF intervention, as shown in Figures 3B, C. The results of RT-qPCR showed that compared with those of the normal group, the expression levels of IL-6, IL-1β, TGF-β, MMP3, and MCP1 mRNA increased in kidney of the model group, and the expression levels of IL-6, IL-1β, TGF-β, MMP3, and MCP1 mRNA decreased after AOF intervention, as shown in Figure 3D. In order to observe the immune-inflammatory cell infiltration in the kidney tissue, we again observed the kidney tissue sections after HE staining. The results showed that compared with the normal group, the kidneys in the model group had an increased infiltration of inflammatory cells; after AOF intervention, the number of inflammatory cells was reduced, as shown in Supplementary Figure 1A; IL-1β, IL-6 are secreted proteins, therefore, we detected their expression levels in renal tissues by the elisa kit. Compared with the normal group, IL-1β, IL-6 levels were significantly increased in the model group; after AOF intervention, IL-1β, IL-6 levels were significantly decreased compared with the model group, as shown in Supplementary Figures 1B, C.

### 3.3 AOF-containing serum ameliorates senescence of HK-2 cells under high glucose intervention

**Figure 4:**
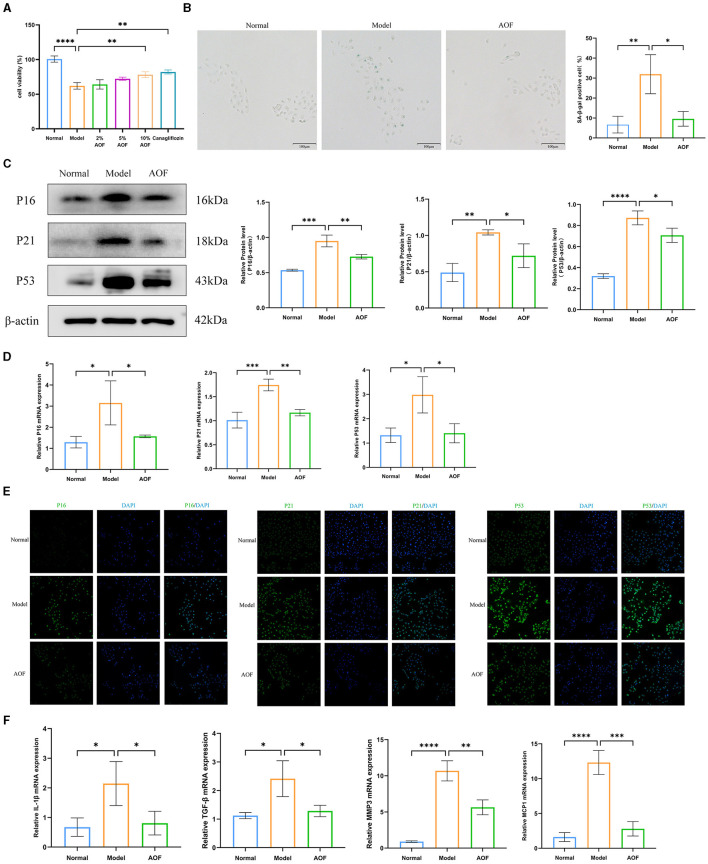
AOF-containing serum ameliorates senescence of HK-2 cells under high glucose intervention. The effect of different doses of AOF on HK-2 cell viability **(A)**; The representative images of SA-β-gal stained in HK-2 cells **(B)**; P16, P21, P53 protein expression levels **(C)**; P16, P21, P53 mRNA expression levels **(D)**; Immunofluorescence staining images of P16, P21, P53 **(E)**; IL-1β, TGF-β, MMP3, MCP1 mRNA expression levels **(F)**. *P < 0.05, **P < 0.01, ***P < 0.001, ****P < 0.0001.

To determine the protective effect of AOF-containing serum on HK-2 cells, we investigated the effect of AOF on glucose-induced viability of HK-2 cells using the CCK8 assay, and found that 10% AOF-containing serum significantly improved the protective effect of high glucose on HK-2 cells. Therefore, this dose was chosen for all subsequent experiments, as shown in Figure 4A. SA-β-gal staining showed that the percentage of blue-stained cells in the normal group was lower than that in the model group, and the percentage of blue-stained cells in the model group was higher than that in the AOF group, as shown in Figure 4B. The results of RT-qPCR and western blot showed that the expression levels of P16, P21, P53 mRNA, and protein in HK-2 cells in the normal group were lower than those in the model group, while the expression levels of P16, P21, P53 mRNA, and protein in the model group were higher than those in the AOF group, as shown in Figures 4C, D. Immunofluorescence staining showed that the fluorescence intensity of P16, P21, and P53 in HK-2 cells of the normal group was lower than that of the model group, while the fluorescence intensity of P16, P21, and P53 in HK-2 cells of the model group was higher than that of the AOF group, as shown in Figure 4E. In addition, the expression levels of IL-1β, TGF-β, MMP3, and MCP1 mRNA in HK-2 cells in the model group were elevated compared with those in the normal group, whereas the expression levels of IL-1β, TGF-β, MMP3, and MCP1 mRNA in the AOF-intervention were reduced, as shown in Figure 4F.

### 3.4 Identification of AOF compounds absorbed into serum

**Figure 5:**
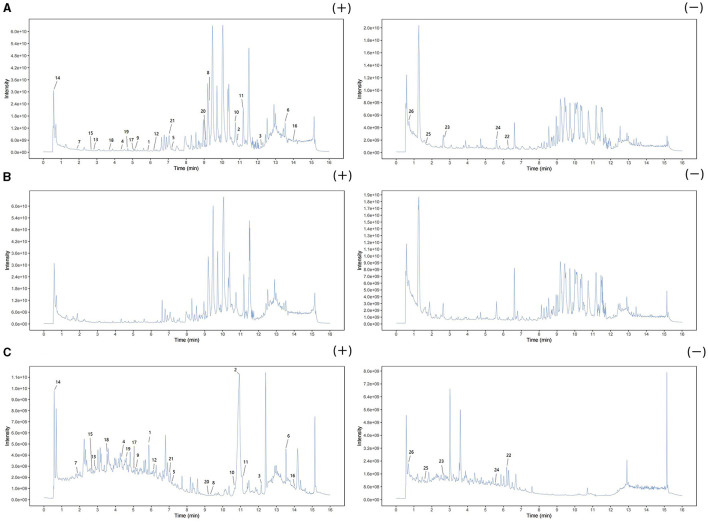
Positive and negative ion chromatograms of AOF-containing serum **(A)**, blank serum **(B)**, and AOF **(C)**.

Three types of samples (AOF, AOF-containing serum, blank serum) were analyzed using UHPLC-QTOF-MS in both positive and negative ion modes. The current total ion chromatograms of the AOF and serum samples are shown in Figure 5. Substances detected in AOF and AOF-containing serum but not in blank serum were considered to be incoming serum compounds of AOF. The results showed that 26 substances may be considered to be active compounds of AOF. The details of compounds are shown in Table 1.

**Table 1:** Analysis of incoming serum compounds of AOF.

**No**.	**Compounds**	**PubchemCID**	**Ion mode**	**m/z**	**RT(s)**	**ppm**	**Adduct**	**MS2**	**Score**
1	Chalcone base + 3O, 1Prenyl	11099375	Postive	325.1402206	356.116	2.39724190889284	[M+H]+	325.142; 326.142; 83.048; 70.065; 55.054	1
2	Methyl 2-aminobenzoate	8635	Postive	152.0705678	658.311	3.73389681620762	[M+H]+	40.09; 153.127; 54.89; 118.172; 95.086	1
3	Abscisic acid	287291	Postive	265.1433757	731.674	1.41714479183018	[M+H]	265.146; 69.07; 228.658; 95.086; 109.101	0.999182231
4	Dihydrodamascenone	577126	Postive	193.1585628	260.3315	2.91380616404506	[M+H]+	107.085; 71.049; 194.116; 105.07; 175.147	0.990970923
5	(1alpha, 6alpha, 7alphaH)-2,4(15)-Copadiene	5316058	Postive	203.1791497	426.435	0.736628198545337	[M+H]+	203.179; 147.117; 204.181; 109.101; 95.086	0.918340462
6	N-Phenyl-1-naphthylamine	7013	Postive	220.1121839	820.01	0.835672545257825	[M+H]+	220.114; 52.314; 54.615; 59.255; 219.175	0.916831846
7	kaempferol-7-O-hexoside	5480982	Postive	449.1076698	117.043	0.735263724475188	[M+H]+	287.053; 85.029; 448.336; 69.033; 449.18	0.887882231
8	Reichsteins substance S	227112	Postive	347.2186586	561.52	0.983267928001687	[M+H]+	347.219; 228.654; 301.234; 68.746; 348.223	0.884241615
9	2′,6′-dihydroxy-4′-methoxydihydrochalcone	169676	Postive	273.1115034	307.802	1.8183805693325	[M+H]+	153.054; 273.112; 119.085; 131.049; 255.133	0.883810846
10	5-Methoxy-1,7-diphenyl-3-heptanone	5319430	Postive	297.1847981	646.559	2.68562624676457	[M+H]+	297.182; 59.06; 228.65; 279.234; 120.9	0.876388462
11	Norephedrine	4786	Postive	152.1067855	668.802	1.41025854502304	[M+H]+	135.08; 93.07; 107.086; 153.09; 111.081	0.815935923
12	Batatasin IV	181271	Postive	245.1169559	366.03	0.179732657876508	[M+H]+	245.119; 227.105; 212.082; 246.123; 228.11	0.796069923
13	Cubebin	287685	Postive	357.1326918	170.4955	0.863046862052634	[M+H]+	137.06; 357.137; 147.044; 151.039; 327.122	0.787360615
14	cis-Sinapic acid	1549091	Postive	225.0756926	40.89965	3.07730788666164	[M+H]+	147.044; 119.049; 175.038; 95.049; 226.118	0.778958538
15	Matricin	92265	Postive	307.1515697	155.678	1.40098283539124	[M+H]+	307.151; 289.143; 123.081; 308.155; 245.119	0.766552308
16	DL-Coniine	9985	Postive	128.1430936	839.076	0.730656224572211	[M+H]+	128.144; 111.091; 71.059; 70.065; 100.087	0.729827923
17	Ixocarpalactone A	327287	Postive	505.2807462	303.272	1.47682877615428	[M+H]+	505.273; 217.159; 159.117; 215.144; 228.654	0.729316846
18	Lucidenolactone	78384956	Postive	457.2582594	218.549	1.61970978292206	[M+H]+	457.291; 225.134; 110.072; 70.065; 228.654	0.680538923
19	Luvangetin	343582	Postive	281.0804953	277.314	1.79552683111913	[M+Na]+	281.081; 239.068; 253.086; 235.076; 238.064	0.677047308
20	1,4a-dimethyl-9-oxo-7-propan-2-yl-3,4,10,10a-tetrahydro-2H-phenanthrene-1-carboxylic acid	10018535	Postive	337.1773922	548.753	1.16315762870959	[M+Na]+	337.179; 307.164; 63.776; 228.667; 167.875	0.651922538
21	Schizandrin A	23915	Postive	417.2250113	419.768	2.36977764344146	[M+H]	417.222; 228.654; 217.159; 59.107; 265.125	0.627921769
22	Isokobusone	3860435	Negative	221.154511	371.473	2.21117380701095	[M-H]-	221.156; 222.158; 203.145; 59.014; 177.129	1
23	Methyl 4-hydroxycinnamate	5319562	Negative	177.0559226	165.741	0.43732319080131	[M-H]	177.055; 149.06; 133.029; 99.927; 162.034	0.832324615
24	Azuleno(5,6-c)furan-1(3H)-one,4,4a,5,6,7,7a,8,9-octahydro-3,4,8-trihydroxy-6,6,8-trimethyl-	156145	Negative	281.1400827	335.088	3.85106055549328	[M-H]-	281.138; 237.15; 219.14; 59.014; 191.144	0.792854538
25	Ethyl caffeate	5317238	Negative	207.0661878	95.5664	0.906752555968421	[M-H]	93.034; 69.035; 207.067; 101.941; 145.931	0.657485385
26	Coumaroyl Hexoside	14158116	Negative	325.0928986	44.60355	0.312040313550902	[M-H]-	163.041; 119.05; 164.043; 325.094; 120.054	0.650285231

### 3.5 Network pharmacology analysis

#### 3.5.1 Acquisition of potential targets for AOF and DKD

The 2D structures of the active compounds were entered into the SwisstargetPrediction and pharmMapper databases for target prediction. Finally, 748 potential targets corresponding to 26 active compounds were obtained. Detailed information is shown in Supplementary Table 2. Two thousand seventy three disease target genes and 545 phenotypes target genes were obtained by searching the GeneCards (https://www.genecards.org/) and OMIM (https://omim.org/) databases with the keywords “diabetic kidney disease,” “diabetic nephropathy,” “ cellular senescence,” and “aging” to collect target genes related to disease and phenotype. Detailed information is shown in Supplementary Table 3.

#### 3.5.2 Active compounds-therapeutic targets network construction

**Figure 6:**
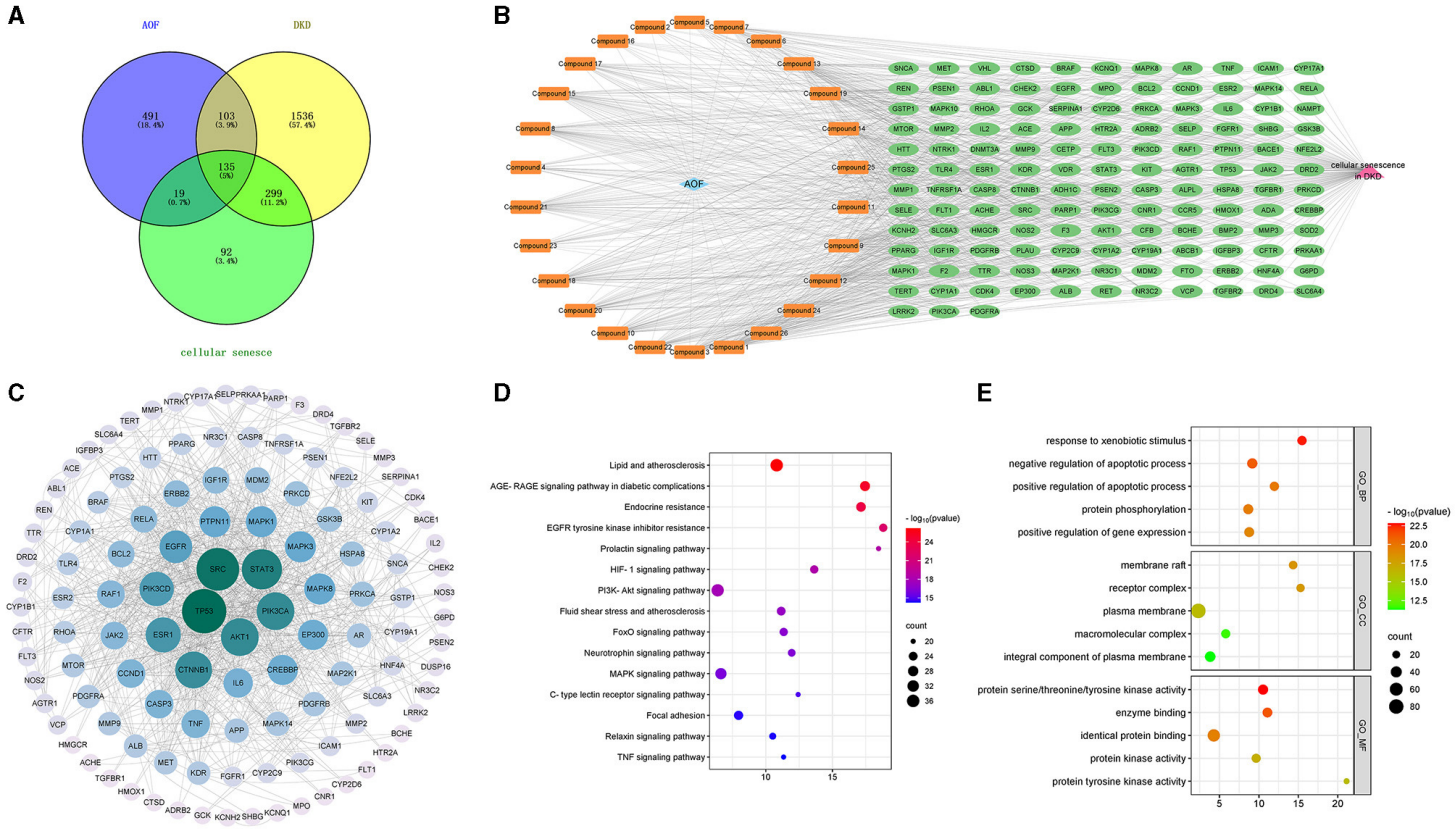
The result of network pharmacology analysis. Venn diagram of AOF, DKD, cellular senescence related targets **(A)**; AOF active compounds-therapeutic targets network, and blue diamond represents AOF, orange round rectangle represents active compound, green ellipse represents therapeutic target, pink triangle represents cellular senescence in DKD **(B)**. PPI network of targets for SQP treatment of DKD. The degree value of this network node is obtained based on the network topology analysis. The more centrally located the node is, the larger the circle is, the greener the color is, the greater the degree value is, indicating that the node is more critical in the overall network **(C)**. The main pathways and biological processes in KEGG enrichment analysis **(D)**. The main pathways and biological processes in GO enrichment analysis **(E)**.

Further taking the intersection of the targets of AOF, DKD and cellular senescence, 135 potential therapeutic targets were obtained as shown in Figure 6A. The active compounds-therapeutic targets network was constructed and the network topology analysis was performed using Cytoscape 3.9.1 software, as shown in Figure 6B and Supplementary Table 4. The topological analysis showed that compound 13 (Cubebin), compound 9 (2′,6′-dihydroxy-4′-methoxydihydrochalcone), compound 1 (Chalcone base + 3O, 1Prenyl), compound 12 (Batatasin IV), and compound 18 (Lucidenolactone) ranked in the top five in terms of degree value size. The larger the degree value, the more critical they are in the network, and thus they are considered to be the core compounds of AOF. Then, the 135 common targets were imported into the STRING database to construct the PPI network. Cytoscape 3.9.1 was used to visualize a protein network relationship graph and perform network topology analysis as shown in Figure 6C, and detailed information is shown in Supplementary Table 5. According to the degree values, the top five key targets of AOF for DKD include TP53, SRC, STAT3, PIK3CA, and AKT1.

#### 3.5.3 GO and KEGG enrichment analysis

To elucidate the biological function of AOF for DKD. The identified target genes were entered into DAVID for GO and KEGG enrichment analysis, using *P* < 0.05 as an indicator of significant biological function. Detailed information is shown in Supplementary Table 6. KEGG pathway enrichment analysis yielded a total of 161 signaling pathways for SQP treatment of DKD. The top 15 pathways selected to remove cancer, tumor, and virus-related pathways are shown in Figure 6D, and these pathways are mainly Lipid and atherosclerosis, Endocrine resistance, EGFR tyrosine kinase inhibitor resistance, and PI3K-Akt signaling pathway. The results of GO enrichment analysis showed that 616 biological processes (BP), 82 cellular components (CC), and 127 molecular functions (MF) were enriched. We selected the top 5 ranked GO terms, as shown in Figure 6E. In the GO_BP category, the terms were mainly involved in response to xenobiotic stimulus, regulation of apoptotic process, protein phosphorylation, regulation of gene expression. In the GO_CC category, the terms mainly included membrane raft, receptor complex, and plasma membrane. In the GO_MF category, the terms mainly consisted of protein serine/threonine/tyrosine kinase activity, enzyme binding, and identical protein binding.

### 3.6 Molecular docking

**Figure 7:**
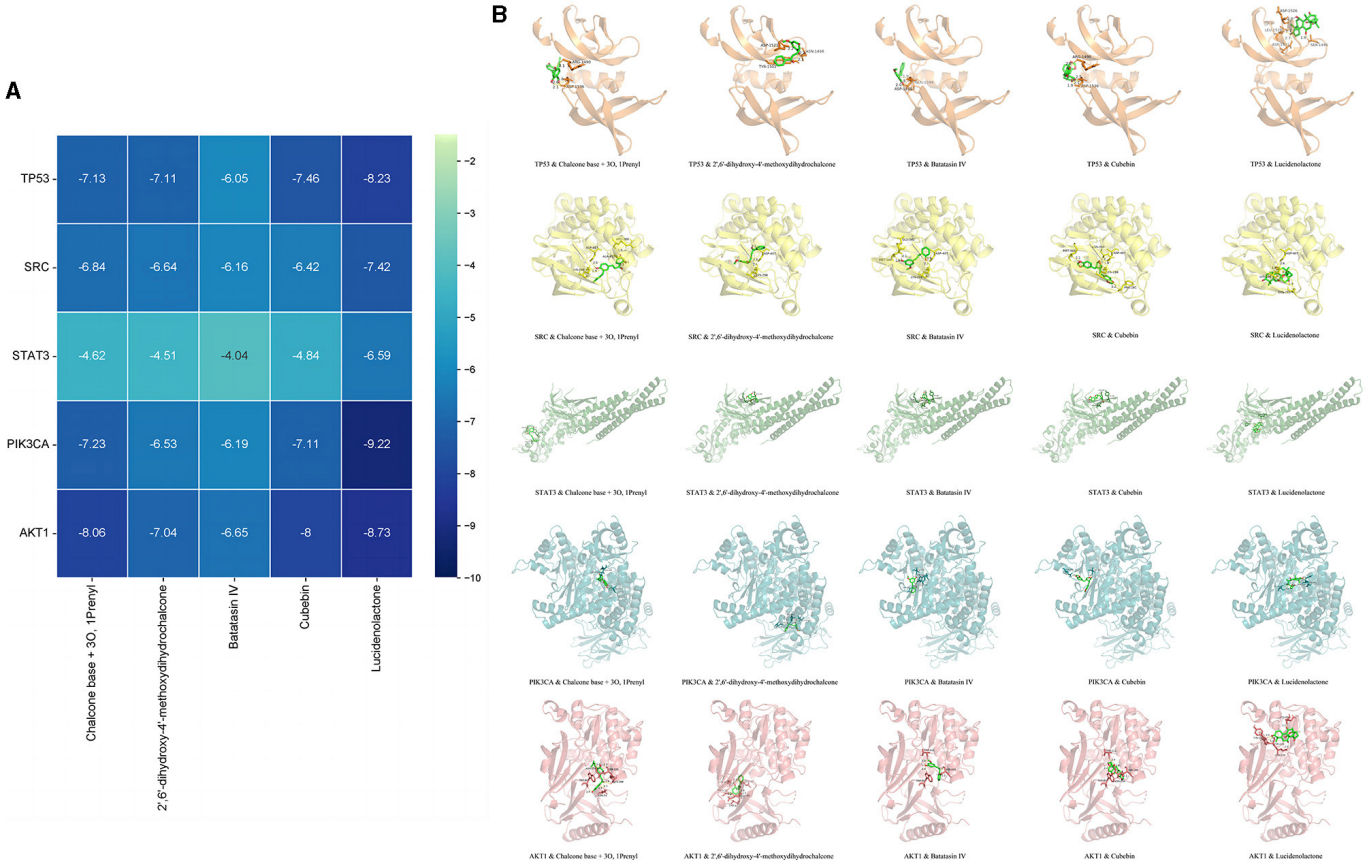
Molecular docking results. Minimum binding energy of each target to AOF bioactive compounds **(A)** and and diagrams of their interactions **(B)**.

Based on the network pharmacological analysis, five core compounds and five targets were selected for molecular docking. The interaction results were plotted in a matrix heat map using binding energy as shown in Figure 7A. When the binding energy was ≤ -1.2 kcal/mol, it indicated that these active ingredients had better binding activity to the protein. Figure 7B shows a schematic representation of the lowest binding energy of the core target to the core compound.

### 3.7 The role of AOF in the regulation of core targets in DKD therapy

#### 3.7.1 *In vivo* experiment

**Figure 8:**
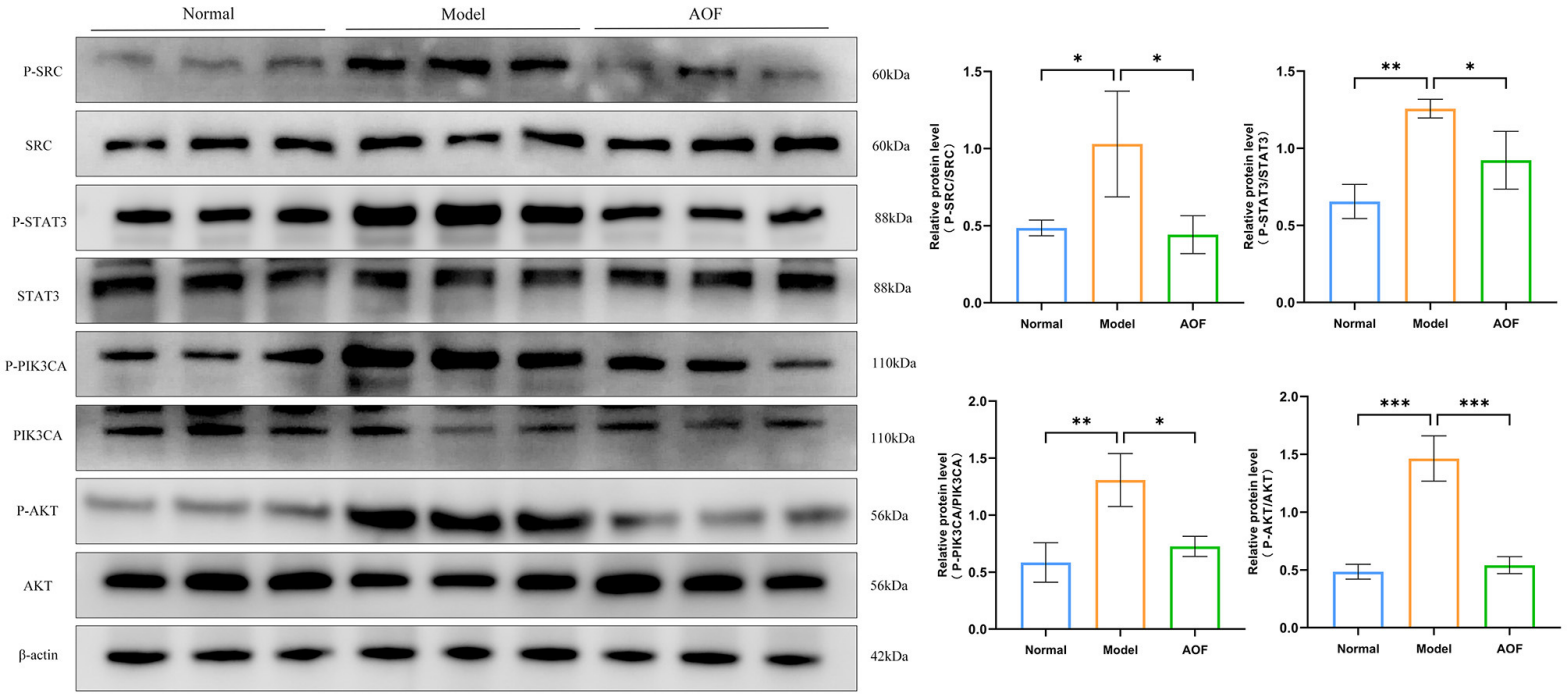
The effect of AOF on the regulation of core targets in kidneys of DKD mice. *P < 0.05, **P < 0.01, ***P < 0.001.

Compared with the normal group, the expression levels of P-SRC/SRC, P-STAT3/STAT3, P-PIK3CA/PIK3CA, and P-AKT/AKT were increased in the kidneys of the mice in the model group; while, after the AOF intervention, the expression levels of P-SRC/SRC, P-STAT3/STAT3, P-PIK3CA/PIK3CA, and P-AKT/AKT were decreased, as shown in Figure 8.

#### 3.7.2 *In vitro* experiment

**Figure 9:**
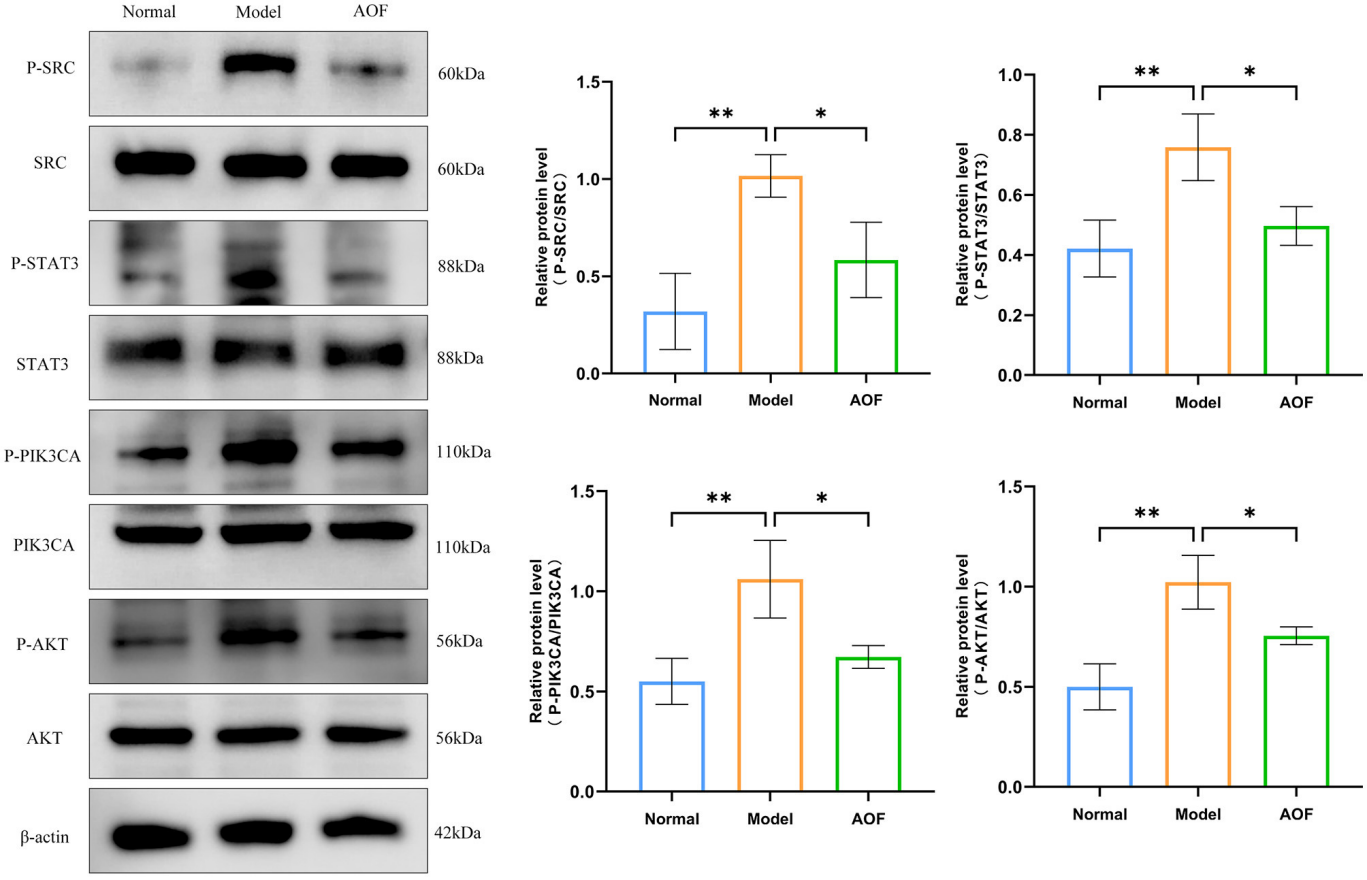
The effect of AOF-containing serum on the regulation of core targets in HK-2 cells. *P < 0.05, **P < 0.01.

Compared with the normal group, the expression levels of P-SRC/SRC, P-STAT3/STAT3, P-PIK3CA/PIK3CA, and P-AKT/AKT were increased in HK-2 cells in the model group; whereas, after the AOF intervention, the P-SRC/SRC, P-STAT3/STAT3, P-PIK3CA/PIK3CA, and P-AKT/AKT expression levels were decreased, as shown in Figure 9.

## 4 Discussion

Chinese medicine believes that kidney deficiency is the root of aging, and tonifying the kidneys is the cure for anti-aging (22). Modern studies have confirmed the efficacy of tonifying the kidneys against aging. For example, BaZiBuShen not only alleviates cognitive deficits (23), but also affects alterations in testicular morphology and spermatogenesis in aging mice (24). Chongcao-Shencha attenuates D-galactose-induced hepatic and renal injury in aging mice (25). Zuogui Wan was able to delay the senescence of bone marrow mesenchymal stem cells (26).

The main feature of senescent organs is the accumulation of senescent cells, and cellular senescence, a permanent state of cell cycle arrest induced by a variety of stresses, is a fundamental aging mechanism. This cell cycle arrest is mainly initiated through these two signaling pathways p53-p21 and p16-RB and maintains growth arrest. Therefore, the expression levels of cell cycle inhibitors P16, P21 are often used as cellular senescence related markers (27). Cellular senescence is one of the important pathogenic mechanisms of DKD. Numerous studies have found that renal cellular senescence improves in conjunction with DKD treatment. For example, metformin improves the senescence of renal tubular epithelial cells in a high-glucose state (28) and preventive effect of Shenkang injection against high glucose-induced senescence of renal tubular cells (29).

Although DKD has traditionally been recognized as a predominantly glomerular disease, current studies report that tubular epithelial cells are more closely associated with deterioration of renal function than glomerular endothelial cell lesions (30). The molecular characteristics of proximal tubular growth in the diabetic kidney are unique and include elements of cell proliferation, hypertrophy, and cellular senescence, and the study of the molecular pathways involved in tubular growth in the diabetic kidney may provide a basis for therapeutic approaches to kidney injury (31). Therefore, the present study used HK-2 cells as an *in vitro* model, as well as db/db mice, which is a spontaneous T2DM animal model, to investigate the efficacy of tonifying the kidneys and the mechanism of action of AOF in the treatment of cellular senescence in DKD. In this study, we found that AOF was able to reduce GLU, mAlb, Scr, BUN, MDA, SOD, and renal pathological tissue damage in DKD mice, reduce the expression of P16, P21, P53 mRNA, and protein, and reduce the expression of SASP (IL-6, IL-1β, TGF-β, MMP3, and MCP1) mRNA. In addition, since immune-inflammatory infiltration is a key factor in inducing the acquisition of a cellular senescence phenotype, we observed the presence of immune-inflammatory cell infiltration in the renal tissues of the mice in the model group by HE staining, and elisa also detected an increase in the levels of IL-6, IL-1β in the renal tissues; whereas, AOF could ameliorate the immune-inflammatory infiltration in the kidneys of the mice with DKD, and reduce the levels of inflammatory factor expression. The *in vitro* experiments showed that AOF-containing serum was able to protect HK-2 cells from the viability impairment caused by high glucose stress, reduce β-galactosidase activity and the expression of cellular senescence-related markers. These results suggest that AOF can ameliorate renal cellular senescence during the progression of DKD and protect renal function and pathological tissue damage. However, its active ingredients and mechanism of action need to be further explored.

We first screened 26 serum-entry compounds of AOF using UHPLC-QTOF-MS and considered them as active compounds that exert pharmacological effects. Then, by constructing an active compound-therapeutic target network and combining it with topological analysis, we screened five compounds including Cubebin, 2′,6′-dihydroxy-4′-methoxydihydrochalcone, Chalcone base + 3O, 1Prenyl, Batatasin IV, Lucidenolactone, and defined them as core compounds for the treatment of cellular senescence in DKD. Cubebin is a dibenzyl butyrolactone lignan with a variety of pharmacological activities such as antibacterial (32), analgesic, anti-inflammatory (33), and attenuated scopolamine-induced elevation of brain acetylcholinesterase activity and oxidative stress levels, with neuroprotective effects (34). 2′,6′-dihydroxy-4′-methoxydihydrochalcone reduced STZ-induced memory deficits and oxidative stress with antioxidant and neuroprotective effects (35) and was found to possess anti-inflammatory activity by regulating the secretion of inflammatory proteins in macrophages and blocking the cleavage of CD62L in neutrophils (36). Chalcone base + 3O, 1Prenyl is also known as licoagrochalcone A. It has been found that licoagrochalcone A possesses pharmacological effects such as anti-inflammatory (37), antiplasmodial (38), and protects against carbon tetrachloride- and acetaminophen-induced HepG2 cell damage (39). Although these compounds have not been identified for use in the treatment of DKD, they may be effective compounds for the treatment of DKD as core compounds for the treatment of cellular senescence in DKD, and their potential pharmacological activities also need to be further investigated.

In order to explore the potential mechanism of AOF in treating cellular senescence in DKD, we identified TP53, SRC, STAT3, PIK3CA, and AKT1 as the core targets for treating cellular senescence in DKD by constructing a PPI network in combination with topological analysis. KEGG enrichment analysis identified EGFR tyrosine kinase inhibitor resistance, PI3K-Akt as an important signaling pathway. GO enrichment analysis identified protein phosphorylation as an important biological process.

After successfully constructing DKD mice model and HK-2 cells model under high glucose stress and elucidating the effect of AOF in the treatment of DKD cellular senescence, we performed molecular docking simulation and experimental validation of the predicted results of network pharmacology. The molecular docking results showed that the above five core compounds had good binding ability to the five key targets. *In vivo* and *in vitro* experiments demonstrated that AOF was able to reduce the expression of TP53 and the phosphorylation levels of SRC, STAT3, PIK3CA, and AKT.

TP53, also known as P53, whose activation leads to cell cycle arrest, senescence or initiation of programmed cell death, is widely recognized as one of the biomarkers of cellular senescence (40). P53 can play a key role in maintaining genome integrity through its role in the DNA damage response (41). In addition, P53 has the ability to regulate the production and secretion of multiple bioactive factors by senescent cells (42). It was found that P53 is activated in various DKD models as well as in human kidneys with DM, and that knocking down P53 in renal proximal tubules reduces renal hypertrophy and prevents the decline of renal function in DKD patients (43). SRC, a membrane-associated non-receptor tyrosine kinase, can be activated by multiple stimuli in the diabetic environment (44). SRC expression levels were found to be upregulated in glomerular and mesangial cells of diabetic rats (45). In addition, SRC-mediated phosphorylation of tyrosine residues activated STAT3 (46). Aberrant activation of STAT3 in DKD may lead to elevated inflammatory responses in renal tissues and increased risk of cellular senescence (5, 47). Therefore, this signaling between SRC and STAT3 plays an important role in maintaining normal cell function and physiological homeostasis. PIK3CA, as an isoform gene in the PI3K family, catalyzes the phosphorylation of phosphatidylinositol, which activates the downstream AKT (48). PIK3CA/AKT signaling was found to modulate multiple downstream cellular signals and targets during DKD progression, affecting the physiological state of renal cells. Abnormal activation of the PI3K/AKT signaling pathway, accompanied by increased oxidative stress, apoptosis, and cell cycle disruption, has been found to occur during senescence of myeloid and pancreatic β-cells (49, 50). Paeoniflorin down-regulates phosphorylation of PIK3CA and AKT and protects podocytes in patients with DKD (51). These findings are similar to ours, suggesting that inhibition of P53 and inhibition of the SRC/STAT3, PIK3CA/AKT signaling pathway may affect the progression of cellular senescence in DKD.

In conclusion, the present study was based on UHPLC-QTOF-MS combined with a network pharmacology approach to predict the core compounds, potential mechanisms of AOF for the treatment of cellular senescence in DKD, and to validate the prediction results by molecular docking and *in vitro* and *in vivo* experiments. The findings laid a preliminary theoretical foundation for further experimental studies. However, due to the complex composition of AOF, its exact active compounds and mechanisms for the treatment of DKD cellular senescence still need to be further verified by extensive pharmacological studies.

## 5 Conclusion

Our study identified the active compounds of AOF and its important compounds and mechanism of action in the treatment of cellular senescence in DKD, and showed that AOF may delay the progression of cellular senescence in DKD by inhibiting TP53 as well as inhibiting the phosphorylation of SRC, STAT3, PIK3CA, and AKT, which provides a modern theoretical basis for the application of AOF in the treatment of DKD.

## Data availability statement

The original contributions presented in the study are included in the article/Supplementary material, further inquiries can be directed to the corresponding authors.

## Ethics statement

The animal study was approved by the Animal Care and Ethics Committee of Hainan Medical University (approval ID: HYLL-2021-389). The study was conducted in accordance with the local legislation and institutional requirements.

## Author contributions

ZY: Conceptualization, Methodology, Writing—original draft, Writing—review & editing. LZ: Data curation, Project administration, Writing—review & editing. YK: Validation, Writing—review & editing. SL: Data curation, Writing—review & editing. XL: Data curation, Writing—review & editing. LL: Writing—review & editing, Data curation. KR: Software, Writing—review & editing. MX: Conceptualization, Project administration, Resources, Supervision, Writing—review & editing. YX: Conceptualization, Funding acquisition, Project administration, Writing—review & editing.
