# Supplementary figures and images for: Integrating serum pharmacochemistry and network pharmacology to explore potential compounds and mechanisms of Alpiniae oxyphyllae fructus in the treatment of cellular senescence in diabetic kidney disease
Source: Front Med (Lausanne). 2024 Jul 3;11:1424644. doi: 10.3389/fmed.2024.1424644 (PMC11251962; doi:10.3389/fmed.2024.1424644)

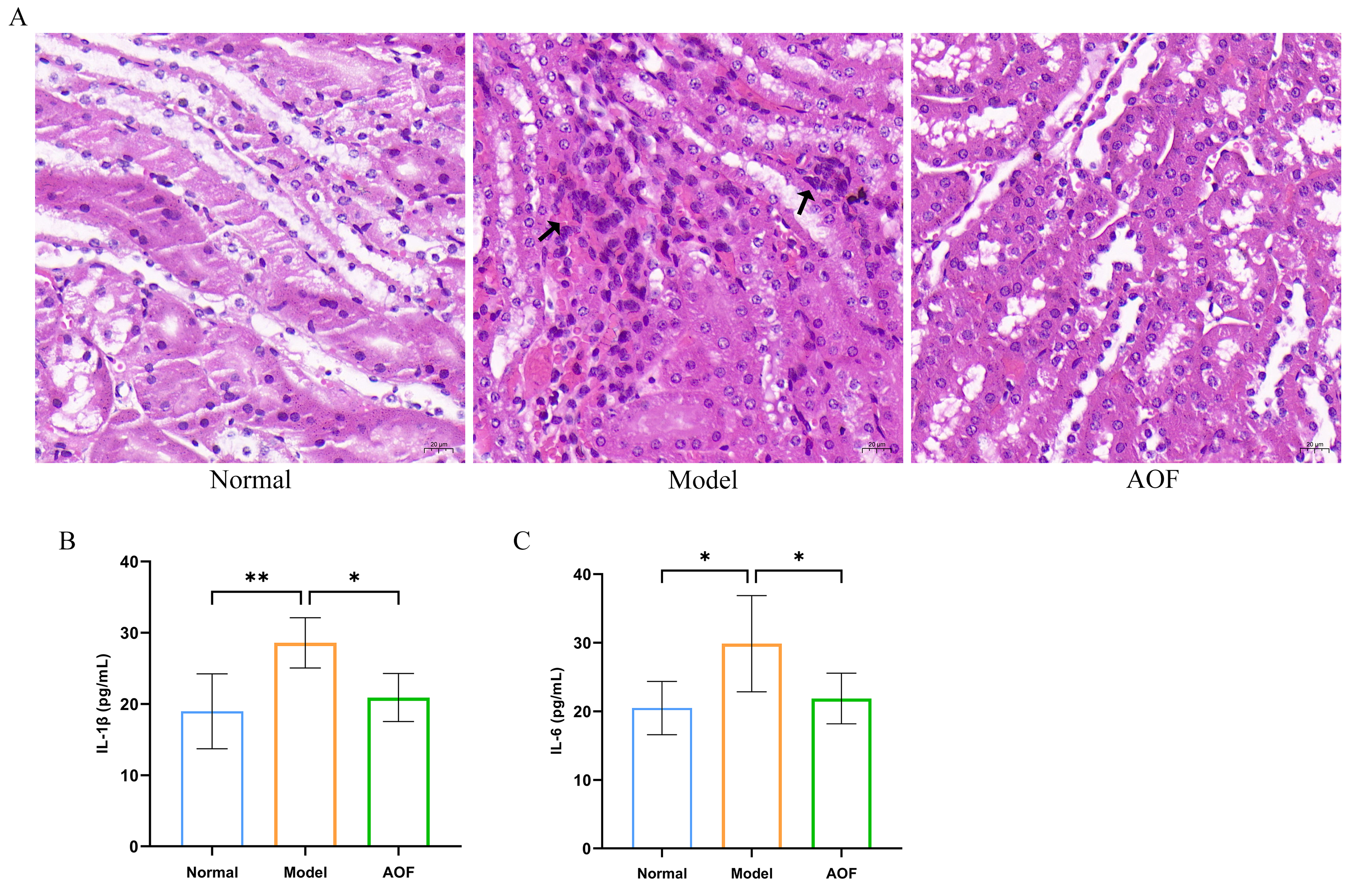

Supplement: Supplementary file 7 [file Image_1.JPEG]
